# KIF3C: an emerging biomarker with prognostic and immune implications across pan-cancer types and its experiment validation in gastric cancer

**DOI:** 10.18632/aging.205694

**Published:** 2024-03-28

**Authors:** Qiangqiang Zhong, Wenbo Hong, Lina Xiong

**Affiliations:** 1 Department of Gastroenterology, Liyuan Hospital, Tongji Medical College, Huazhong University of Science and Technology, Wuhan 430077, China; 2 Laboratory of Metabolic Abnormalities and Vascular Aging Huazhong University of Science and Technology, Wuhan 430077, China

**Keywords:** KIF3C, pan-cancer, gastric cancer, biomarker, prognosis

## Abstract

Kinesin Family Member 3C (KIF3C) assumes a crucial role in various biological processes of specific human cancers. Nevertheless, there exists a paucity of systematic assessments pertaining to the contribution of KIF3C in human malignancies. We conducted an extensive analysis of KIF3C, covering its expression profile, prognostic relevance, molecular function, tumor immunity, and drug sensitivity. Functional enrichment analysis was also carried out. In addition, we conducted *in vitro* experiments to substantiate the role of KIF3C in gastric cancer (GC). KIF3C expression demonstrated consistent elevation in various tumors compared to their corresponding normal tissues. We further unveiled that heightened KIF3C expression served as a prognostic indicator, and its elevated levels correlated with unfavorable clinical outcomes, encompassing reduced OS, DSS, and PFS in several cancer types. Notably, KIF3C expression exhibited positive associations with the pathological stages of several cancers. Moreover, KIF3C demonstrated varying relationships with the infiltration of various distinct immune cell types in gastric cancer. Functional analysis outcomes indicated that KIF3C played a role in the PI3K-AKT signaling pathway. Drug sensitivity unveiled a positive relationship between KIF3C in gastric cancer and the IC50 values of the majority of identified anti-cancer drugs. Additionally, KIF3C knockdown reduced the proliferation, migration, and invasion capabilities, increased apoptosis, and led to alterations in the cell cycle of gastric cancer cells. Our research has revealed the significant and functional role of KIF3C as a tumorigenic gene in diverse cancer types. These findings indicate that KIF3C may serve as a promising target for the treatment of gastric cancer.

## INTRODUCTION

Gastric cancer (GC) has emerged as the fourth most prevalent malignant tumor globally, according to [1]. Unfortunately, a substantial number of GC patients receive their diagnosis in the advanced stages of the disease, resulting in a significant number of cancer-related fatalities. More than half of all gastric cancer cases are reported in developing nations, particularly in high-incidence regions such as East Asia and South America [2]. Gastric cancer is characterized by its insidious onset, rapid progression, and a propensity for postoperative recurrence, rendering it one of the most pressing public health challenges in China. The limited rate of early diagnosis in gastric cancer is attributed not only to its high invasiveness but also to the absence of specific biomarkers for early detection. In light of this situation, numerous researchers are actively investigating novel diagnostic markers and potential therapeutic targets for this disease.

Given the substantial utility of molecular targeted therapy in gastrointestinal tumors, investigating the molecular biology traits of gastric cancer cells holds immense importance in the quest for new therapeutic targets and the enhancement of gastric cancer diagnosis and treatment [3]. Kinesin motor proteins constitute a crucial class of molecular motor proteins within organisms, actively involved in substance transport. These proteins harness the energy released through ATP hydrolysis to perpetuate their movement along microtubules, facilitating the transportation of various cargo molecules. This process serves as the driving force behind the movement of membrane organelles, protein complexes, mRNAs, and more [4].

Presently, over 45 types of kinesin proteins have been identified, and based on their structural attributes, they can be categorized into 14 kinesin subfamilies. Notably, the kinesin-2 subfamily is recognized as the primary motor protein engaged in intraflagellar transport [5]. Within the kinesin-2 family, notable members include KIF3A, KIF3B, KIF3C, and KIF17. These molecular motor proteins, belonging to the kinesin-2 family, are instrumental in the intracellular transport of diverse protein complexes and vesicles, exerting pivotal roles in numerous biological processes. In recent years, it has come to light that kinesin-2 plays a critical role in intraflagellar transport, and impairments in its function can give rise to a spectrum of diseases, including cancer.

Kinesin family member 3C (KIF3C) is associated with diverse roles in cellular processes such as antigen processing, presentation, vesicle transport, and retrograde transport from the Golgi apparatus to the endoplasmic reticulum [6]. Notably, there is accumulating evidence highlighting the regulatory role of KIF3C in cancer. For instance, KIF3C demonstrates increased expression in prostate cancer, where it has been revealed to enhance the development of prostate cancer [7]. Moreover, elevated levels of KIF3C have been associated with an adverse prognosis in NSCLC. Both *in vitro* and *in vivo* experiments have provided confirmation of its role in promoting the malignant behavior of NSCLC [8].

KIF3C has also been identified as a factor that can impede glioma growth by modulating the PI3K/AKT/mTOR pathway, thereby extending the survival of glioma patients, and offering a prospective target for glioma treatment [9]. Subsequent research by [10] and colleagues corroborated the notion that KIF3C overexpression fosters the malignant progression of glioma cells while hindering apoptosis. Their findings hint at the involvement of the PI3K/AKT pathway and epithelial-mesenchymal transition (EMT) in the mechanisms through which KIF3C governs these functions. Similarly, the downregulation of MiR-2053 has been shown to elevate KIF3C expression and activate the PI3K/AKT pathway, thereby contributing to the malignant progression of esophageal cancer cells [11]. However, there is presently a scarcity of research pertaining to the potential molecular mechanisms and gene functions of KIF3C in gastric cancer. In summary, KIF3C exhibits itself as a promising candidate for both a tumor marker and a prospective therapeutic target. Nonetheless, the prevalence of high KIF3C expression in cancer cells and its underlying biological functional mechanisms in tumors remain uncertain.

Utilizing data from publicly available databases, we conducted a comprehensive investigation into the differential expression of KIF3C across various cancer types. Employing a multidimensional analysis, we assessed the independent prognostic value of KIF3C in gastric cancer (GC). In an effort to delve deeper into the functional network and pathogenic mechanisms associated with KIF3C in GC, we implemented KIF3C gene expression knockdown in gastric cancer cell lines characterized by high KIF3C expression. We observed the resultant impact on various biological functions, encompassing proliferation, migration, invasion, apoptosis, and cell cycle distribution within the tumor cells. Our research has bridged a critical knowledge gap, shedding light on the biological functions of KIF3C and highlighting its potential as a promising prognostic biomarker for GC.

## MATERIALS AND METHODS

### KIF3C data acquired and expression analyses

The transcriptome and clinical data from 33 different cancers were obtained by downloading information from the TCGA database (https://portal.gdc.cancer.gov/) through UCSC Xena (https://xena.ucsc.edu/). Additionally, we employed the expression of KIF3C at the single cell level through the TISCH. Cancer Cell Line Encyclopedia (CCLE) database, which is accessible at (https://sites.broadinstitute.org), to acquire mRNA expression levels of KIF3C in cancer cell lines. All expression data were standardized through log2 conversion. Furthermore, we employed qPCR and the GEO database to elucidate the differential expression of KIF3C in gastric cancer relative to normal gastric tissues.

### Prognostic potential analysis of KIF3C

The prognostic potential of KIF3C was evaluated using survival analysis by stratifying the samples into KIF3C high and low groups based on a median value. Next, survival analyses were conducted to assess the prognosis related to varying KIF3C expression in 33 types of cancer, employing the “survival” and “survminer” packages. OS, DSS, and PFS were employed to appraise the prognostic significance of KIF3C.

### Correlation analysis of KIF3C and clinicopathological characteristics

Categorization of cancer cases into two subgroups, denoted as the “KIF3C low group” and the “KIF3C high group,” was performed according to the median expression value of KIF3C. The examination of the correlation between KIF3C and various clinical phenotypes across multiple tumors was conducted, and the outcomes were visually represented utilizing R packages “limma” and “ggpubr”.

### Assessment of KIF3C diagnostic value

To evaluate the diagnostic potential of KIF3C, we conducted ROC curve analysis using the “pROC” and “ggplot2” R packages. An AUC greater than 0.9 signifies a high degree of diagnostic accuracy.

### Differentially expressed genes (DEGs) identified and functional enrichment analysis in gastric cancer

We utilized the “limma” package to identify the DEGs between KIF3C expressed at high and low levels in gastric cancer patients. Next, we performed Gene Ontology (GO) analyses to categorize these genes into various functional categories, encompassing BP, MF, and CC. Furthermore, KEGG pathway analyses of the DEGs were executed. In our study, we utilized the clusterProfiler package [12] for conducting GO and KEGG analyses.

### Correlation between KIF3C and immune cell infiltration, immune checkpoints, and immunotherapy response estimation

The ESTIMATE algorithm was utilized to calculate the stromal scores (SSs), ESTIMATE scores (ESs) and immune scores (ISs) of each tumor tissue in TCGA. Then, the correlations of KIF3C with SSs, ISs and ESs were analyzed. The CIBERSORT ssGESA approach was applied to analyze the proportions of 24 TIICs via a normalized gene expression matrix. Meanwhile, the Wilcox test was applied to compare the immune infiltration differences between the KIF3C high- and low-expression groups. The correlations between KIF3C and immune checkpoints were visualized through a scatter plot. We also investigated the correlation between KIF3C mRNA expression and MSI using Spearman’s correlation test. The Cancer Immunome Atlas (TCIA) (https://tcia.at/) was used to download the immunophenoscores (IPS) of STAD. The relationship between KIF3C and IPS was analyzed to predict immunotherapy sensitivity. Tumor immune dysfunction and exclusion (TIDE) score was calculated via TIDE algorithm to infer patients’ responses to immune checkpoint blockade (ICB) treatment, whose main targets are PD-L1, PD-1, and CTLA4, and these analyses were conducted employing R software version 4.0.3.

### Drug sensitivity analysis in gastric cancer

We employed the GDSC gene expression and drug sensitivity datasets, which were made available through the R package “oncoPredict,” as our training data. Subsequently, we utilized the STAD-TCGA expression dataset as our test data. To assess drug sensitivity among the samples, we utilized the “calcPhenotype” function. Following this, we combined the KIF3C gene expression data with the drug sensitivity data and conducted a Wilcoxon rank sum test.

### Cell culture

Human normal gastric epithelial cells (GES-1) and GC (HGC-27, AGS, MKN-45) cell lines were obtained from the Cell Bank of the Chinese Academy of Sciences (Shanghai, China). The HGC-27 and MKN-45 cell lines were grown in RPMI1640 medium (Thermo Fisher Biochemical Products Beijing Corporation, China), and the GES-1 cell line was cultured in DMEM medium (Thermo Fisher Biochemical Products Beijing Corporation, China), while the AGS cell line was grown in F12K medium (BeNa Culture Collection, Beijing, China). The culture medium comprised 10% fetal bovine serum (FBS) sourced from PAN-Biotech Corporation, alongside 100 U/ml penicillin and 100 μg/ml streptomycin from NCM Biotech (Suzhou, China). The cells were cultivated in a humidified environment at 37°C with a 5% CO2 supplement.

### RNA isolation and qRT-PCR

We employed the RNA-easy™ Isolation Reagent from Vazyme Company (Nanjing, China) for total RNA extraction, following the manufacturer’s guidelines. The extracted RNA was subsequently reverse transcribed into complementary DNA (cDNA) using another product from Vazyme Company, which is named HiScript Q RT SuperMix for qPCR reagent Kit. Real-time PCR analysis was then conducted using the SYBR Green detection method. The sequences of qPCR primers are as follows: GAPDH, Forwards: 5’-TGACCTGCCGTCTAGAAAAACCT-3’. Reverse: 5’-GCTGTTGAAGTCAGAGGAGACCA-3’; KIF3C, Forwards: 5’-ACCTGCAGGAACAGAAGGAGC-3’, and KIF3C Reverse: 5’-GCATCTTCTCCTTCTCCTCCA -3’. Relative levels of expression were computed using the 2−ΔΔCt method.

### siRNA transfection

We seeded well-growing HGC-27 and AGS cells in a 6-well plate, and 24 hours later, we performed cell transfection according to the instructions of the Lipofectamine 2000 reagent. Transfection with si-NC established the si-NC group, while transfection with si-KIF3C established the si-KIF3C group. Subsequently, we validated the transfection efficiency using qPCR. The small interfering RNA sequences used in the experiment were as follows: siRNA-KIF3C:(sense, 5′-GGCUAGAGCUGAAAGAGAATT-3′, antisense, 5′-UUCUCUUUCAGCUCUAGCCTT-3’); siRNA-NC: (sense, 5’-UUCUCCGAACGUGUCACGUTT-3′, antisense, 5-ACGUGACACGUUCGGAGAATT-3′).

### CCK-8 assay

We initially plated well-growing HGC-27 and AGS cells in a 96-well plate at a density of 2000 cells per well. After a 24-hour adherence period, we categorized the cells into two groups: one group received si-NC transfection, while the other received si-KIF3C transfection. Subsequently, at 0, 24 hours, 48 hours, and 72 hours post-transfection, we added 10 μL of CCK-8 solution to each well, and subsequently, the cells were incubated at 37°C for 1 hour. Finally, we measured the absorbance (OD value) at 450 nm using a microplate reader.

### Colony formation assay

Following a 24-hour transfection, cells were harvested from 6-well plates and resuspended to create a single-cell suspension. Subsequently, 700 cells were seeded per well in new 6-well plates. To ensure even cell distribution, the plate was gently agitated in a crosswise pattern. Incubation was carried out at 37°C in a 5% CO2 humidified environment, with medium changes as required. Upon reaching an approximate colony size of 50 cells, terminate the experiment, then fix the cells with a 4% paraformaldehyde solution for 30 minutes, and stain them with a 0.1% crystal violet solution for an additional 30 minutes. Subsequently, photographs of the stained cells were taken, and the colony count was determined using Image J software.

### EdU assay

The HGC27 and AGS cells, which had been transfected and were in the logarithmic growth phase, were plated in 96-well plates with a 50μmol/L EdU medium. Following a 2-hour incubation, the cells were gently rinsed with PBS. Subsequently, the cells were fixed using paraformaldehyde. Afterward, 200 μL of a 2 mg/ml glycine solution was added, and the cells were incubated for 5 minutes. Following this, the cells were washed with PBS for 5 minutes on a shaker. Following that, the cells underwent a decolorization treatment using 0.5% TritonX-100, with continuous incubation on a shaker for 10 minutes. Afterward, we performed two PBS washes, each lasting 5 minutes. The cells were subsequently stained with Apollo in a light-protected environment at room temperature for 30 minutes, and this was followed by staining with DAPI staining solution, also in darkness at room temperature, for 20 minutes. Following a final washing step with PBS, images of the cells were captured using a fluorescence microscope.

### Wound-healing assay

The HGC-27 and AGS cells were initially seeded in a 6-well plate and transfected once they reached a confluence of 60-70%. After transfection, when the cell density reached 100%, we created a scratch along the axis of the cell monolayer using a 200 μL pipette tip. The detached cells were subsequently washed three times with PBS, and serum-free basal culture medium was added. The plate was then returned to the cell culture incubator for further incubation. The process of wound healing was observed under an inverted microscope at 0 and again at 48 hours after creating the scratch.

### Transwell assays

We seeded well-growing HGC-27 and AGS cells in a 6-well plate, and after 24 hours, we harvested and counted the cells. We mixed a cell suspension containing 50,000 cells with 200 μL of serum-free basic culture medium, loaded it into the transwell chamber, and subsequently added 500 μL of culture medium containing 20% FBS beneath the chamber. Then the transwell insert was placed in a cell culture incubator. After a 24-hour incubation period, the upper chamber fluid was aspirated, and the cells were subsequently fixed with a 4% paraformaldehyde solution for 30 minutes. Following fixation, the cells were stained with a crystal violet solution for 20 minutes. We observed and photographed the cells under a microscope and counted them. For the invasion assay, 5×10^4 cells were seeded in transwell chambers pre-coated with Matrigel. Then the chamber was loaded with serum-free medium, while a medium containing 20% FBS was submerged beneath the chamber. After 24 hours, the count of cells that had traversed the Matrigel barrier was conducted under a microscope.

### The cell cycle was determined by flow cytometry

We transfected cells in six-well plates for 48 h, digested cells for 2 min without EDTA and collected by centrifugation, washed three times in PBS, adding cooled 70% ethanol solution, blew up into a single cell suspension, and fixed in a -20°C refrigerator. After centrifugation at 1500 rpm for 10 minutes, the residual ethanol was completely removed by three washes with PBS, 600μL PI dye solution was quickly blown, 4°C away from light for 1 h. Red fluorescence and light scattering were assessed using flow cytometry with excitation at 488 nm, measuring the percentage of G0 / G1, S phase and G phase 2 / M phase in each group.

### Apoptosis assay

Digest cells with trypsin without EDTA, centrifuge 100 μL of cell suspension at 4°C, 1000 rpm for 5 minutes, and discard the supernatant. Following the instructions in the Annexin V-FITC/PI apoptosis assay kit (Beyotime Biotechnology, Shanghai, China), we added 400 μL of buffer to each tube, followed by 5 μL of Annexin V-FITC and 5 μL of PI. Subsequently, we incubated cells at room temperature in the dark for 10 minutes, then used a flow cytometer to measure the apoptosis rate of cells in each group.

### Statistical data analysis

This part uses Rstudio (V4.2.1) and GraphPad Prism 10 for statistical analysis. If the measurement data follow a normal distribution, it will be presented as the mean ± standard deviation, and analytical comparisons between the two groups will be conducted using a t-test. Two-tailed t-test was used between comparisons. Non-normally distributed measurement data are expressed using the median ± quartiles, Mann-Whitney U rank sum test for comparisons between two groups and Kruskal-Wallis H test for differences between multiple groups. Analysis: P <0.05.(*P <0.05, **P <0.01, ***P <0.001, ****P <0.0001).

## RESULTS

### mRNA levels of KIF3C in normal and cancer tissues

**Figure 1:**
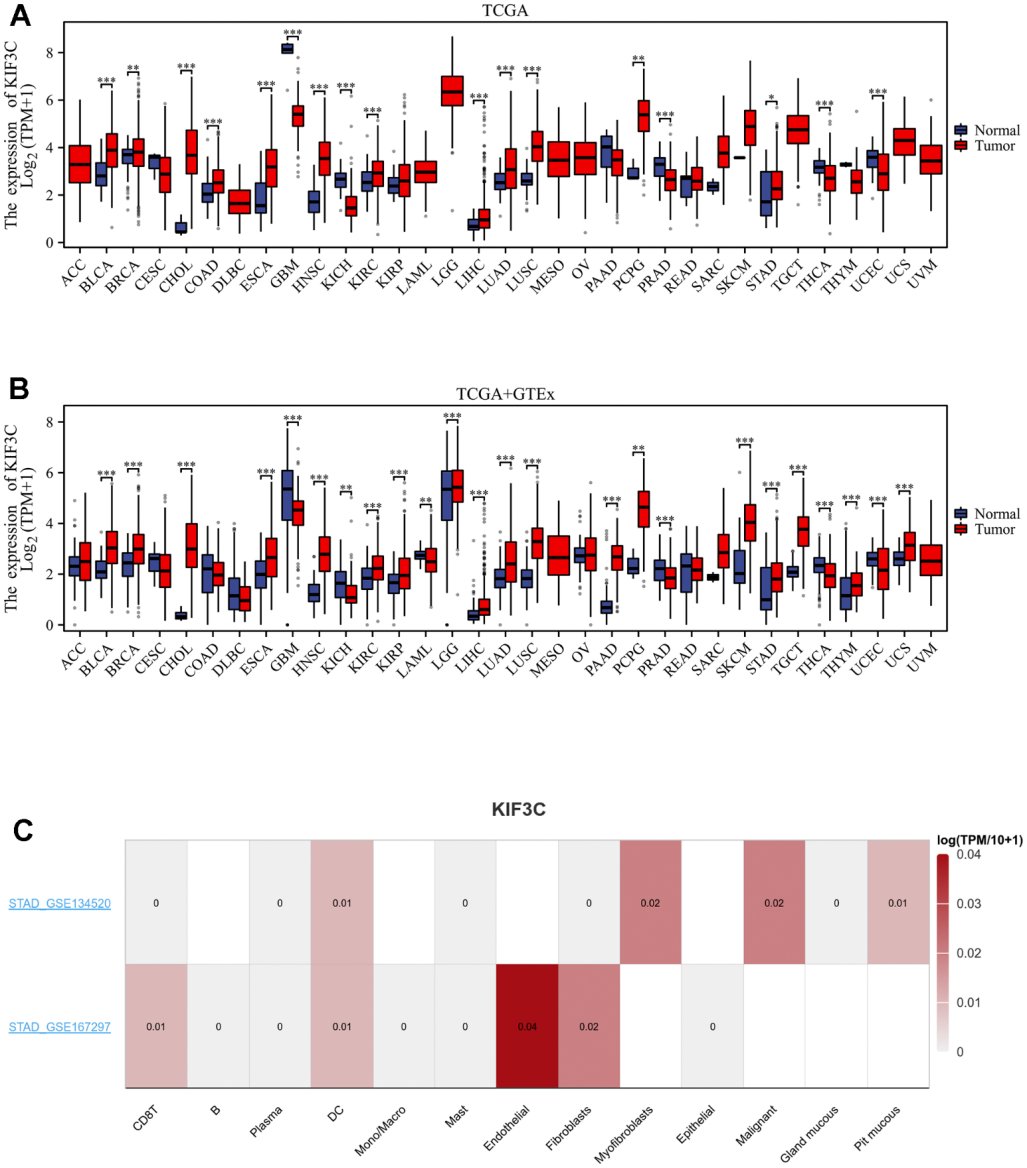
**Expression levels of KIF3C in different tissues in pan-cancer.** (**A**) Expression levels of KIF3C in tumor and paired adjacent noncancerous tissues from TCGA. (**B**) KIF3C expression difference in 33 tumors integrating data of normal tissues in GTEx database and tumor tissues in TCGA database. (**C**) KIF3C expression in immune cells in STAD via the TISCH database. *p < 0.05, **p < 0.01, ***p < 0.001, ns p ≥ 0.05.

In this study, our aim was to assess the expression of KIF3C across diverse tumor types. To discern the specificity of KIF3C expression, our initial approach involved the utilization of data from the TCGA database to investigate variations in KIF3C expression levels. Our analysis revealed a significant increase in KIF3C expression levels in several cancer types, including BLCA, STAD, CHOL, COAD, ESCA, HNSC, KIRC, LIHC, LUAD, LUSC, PCPG, and STAD, when compared to normal tissues (P<0.05) (Figure 1A). The results of TCGA and GTEx consistently showed relatively high KIF3C expression levels in 18 types of cancer, with COAD being the exception (Figure 1B). Moreover, by utilizing TISCH TCGA datasets, we assessed the KIF3C levels in single cells paired tumors and normal samples, uncovering elevated expression in endothelial cells in STAD in only 10 types of cancer tissues (Figure 1C). We also investigated KIF3C mRNA expression in CCLE cancer cell lines (Figure 1D). Additionally, the GEO dataset validated the expression of KIF3C in various cancers, except for PCPG, THCA, and UCEC, and these results were consistent with those from TCGA (Supplementary Figure 1).

### Evaluating the prognostic value of KIF3C

We performed a one-way Cox regression analysis to assess the correlation between KIF3C levels and overall survival (OS) within the TCGA cohort. The forest plots, encompassing the 33 different tumor types, illustrated that elevated KIF3C levels were linked to an increased risk in LIHC, LUAD, MESO, SARC, STAD, and UCEC, while they were associated with a decreased risk in KIRC and LGG (Figure 2A). To further investigate the connection between KIF3C expression and patient outcomes, we employed the KM plotter portal and the logrank method. Kaplan-Meier (KM) analysis unveiled that elevated KIF3C levels were associated with reduced overall survival in LUAD, MESO, STAD, and UCEC, whereas lower KIF3C levels were linked to decreased overall survival in LGG (Figure 2B).

**Figure 2:**
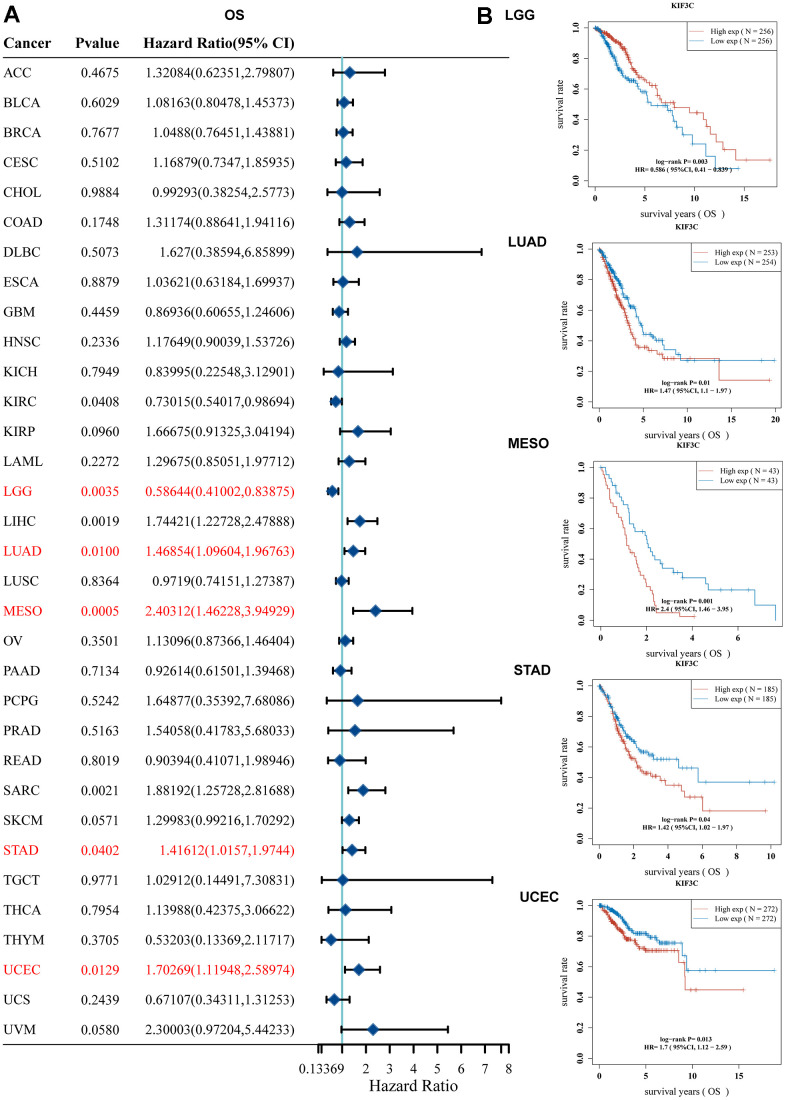
**Correlation between KIF3C expression and survival prognosis.** (**A**) Forest plot of OS correlation in TCGA. (**B**) Kaplan-Meier analysis of the correlation between KIF3C and OS.

Similarly, we examined the relationship between KIF3C levels and Disease-Specific Survival (DSS) among patients. Univariate Cox regression analysis demonstrated a significant impact of KIF3C expression on the Disease-Specific Survival (DSS) of KIRP, LGG, LIHC, LUAD, MESO, SARC, STAD, and UCEC (Figure 2C). Data from the KM plotter portal revealed a significant correlation between highly expressed KIF3C and poor Disease-Specific Survival (DSS) in patients with LUAD, MESO, STAD, and UCEC. Conversely, diminished KIF3C level was significantly correlated with inferior Disease-Specific Survival (DSS) in patients afflicted with LGG (Figure 2D).

**Figure 2:**
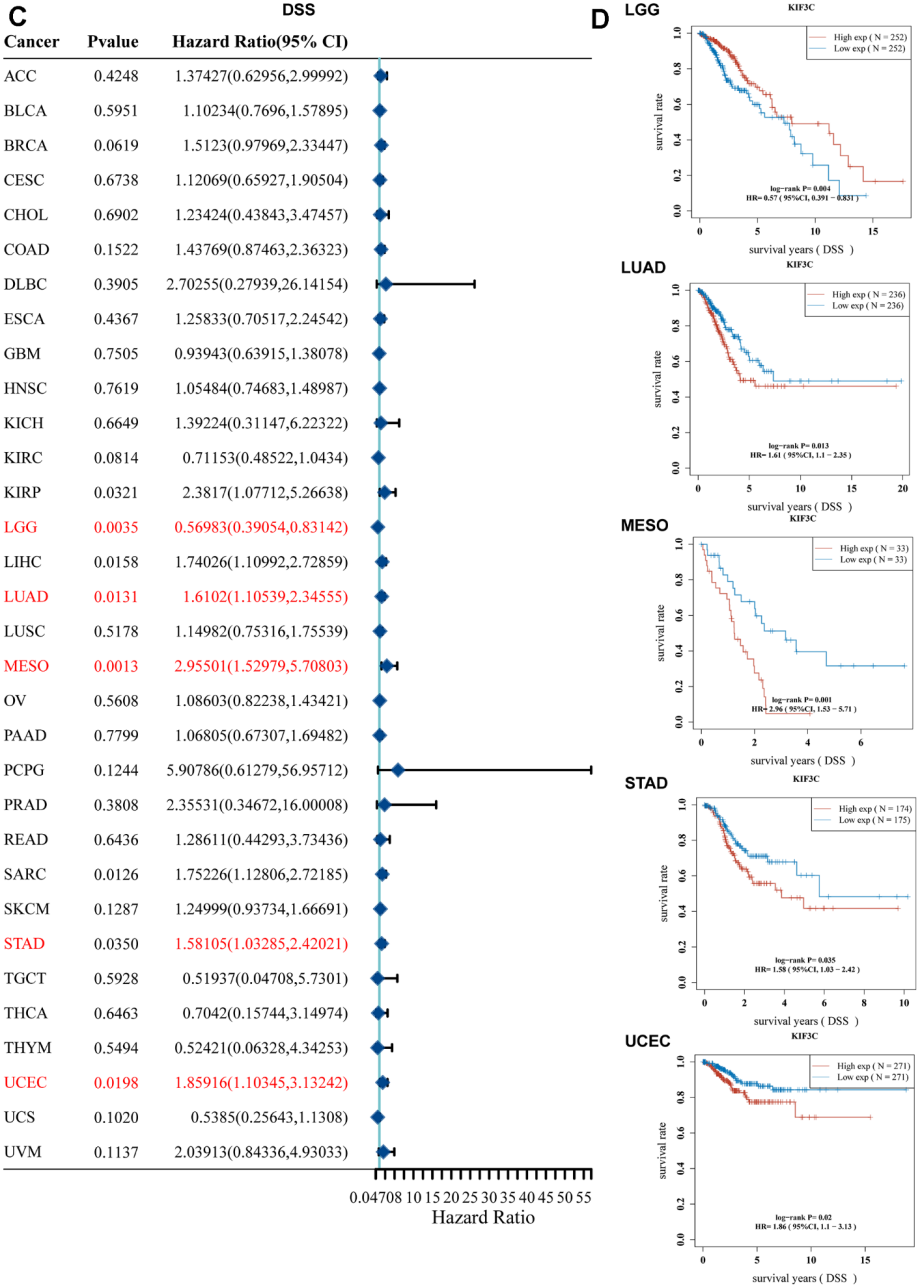
**Correlation between KIF3C expression and survival prognosis.** (**C**) Forest plot of DSS correlation in TCGA. (**D**) Kaplan-Meier analysis of the correlation between KIF3C and DSS.

Moreover, a forest plot visually depicted that KIF3C expression had a notable impact on Progression-Free Survival (PFS) in ACC, STAD, GBM, LGG, LUAD, MESO, PRAD, SARC, STAD, and UCEC (Figure 2E). Furthermore, KM curves confirmed that KIF3C overexpression was correlated with poor PFS in LUAD, MESO, STAD, and UCEC, and low KIF3C expression indicated worse PFS in LGG (Figure 2F). In summary, these findings establish an inverse relationship between the level of KIF3C and the survival duration in specific cancers. Our data suggest that KIF3C holds the potential to function as a prognostic biomarker linked to patients’ OS, DSS, and PFS in various human cancers, particularly in instances of LGG, LUAD, MESO, STAD, and UCEC.

**Figure 2:**
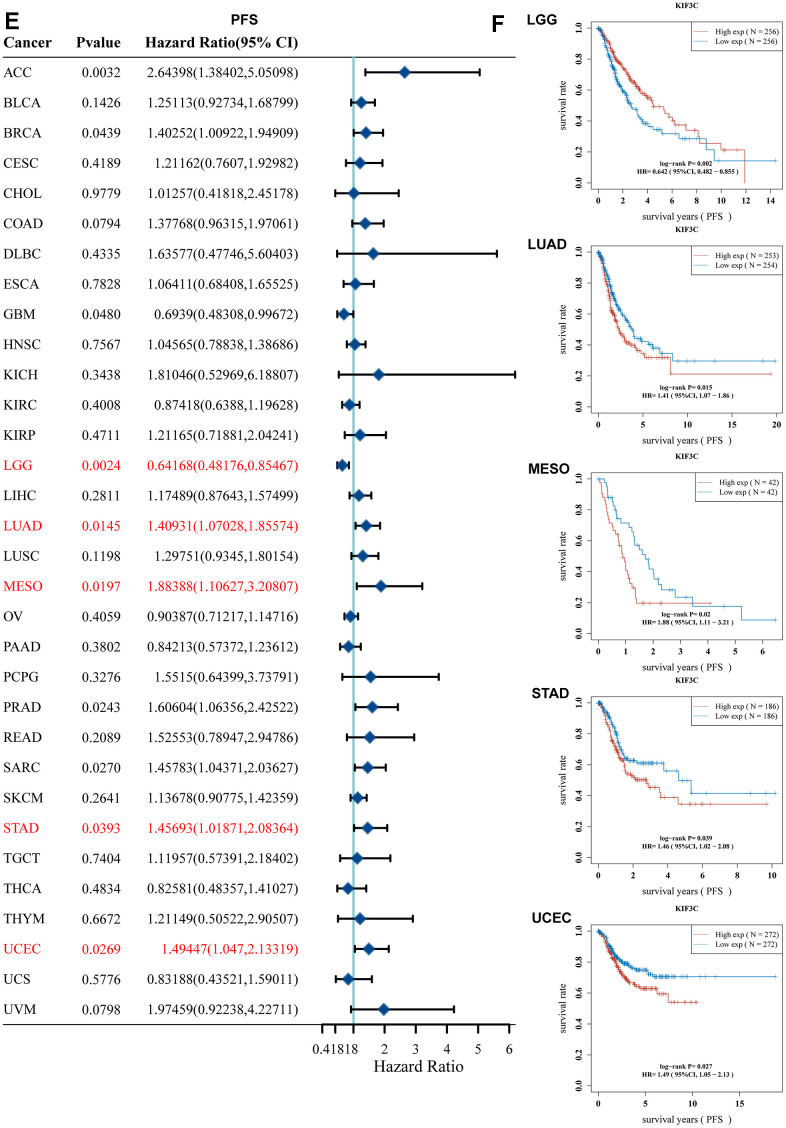
**Correlation between KIF3C expression and survival prognosis.** (**E**) Forest plot of PFS correlation in TCGA. (**F**) Kaplan-Meier analysis of the correlation between KIF3C and PFS.

### Correlation analysis between KIF3C expression and clinicopathological features

**Figure 3:**
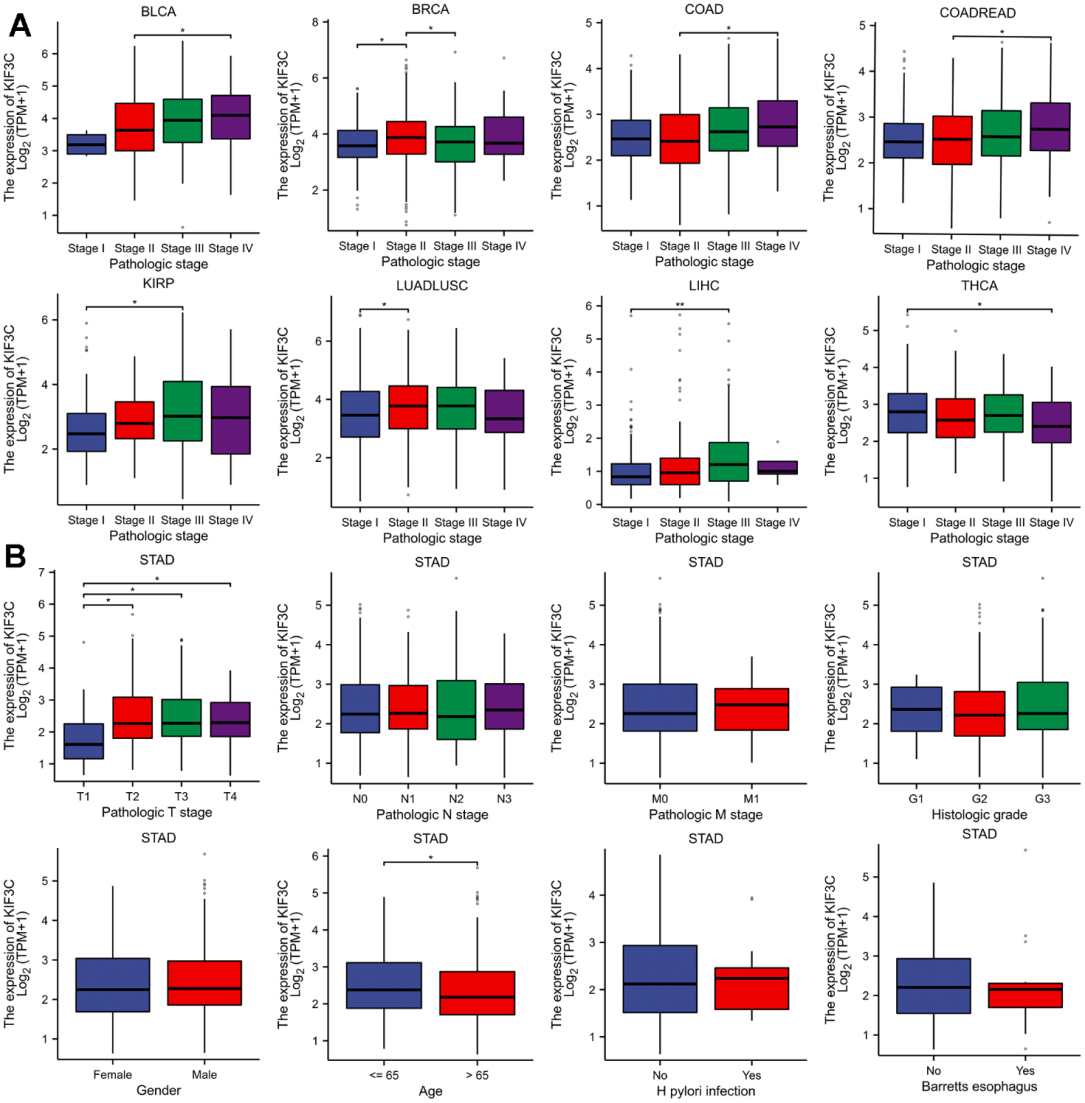
**Correlation between KIF3C and clinical characteristics.** (**A**) Correlation between KIF3C expression and pathological stages of BLCA, STAD, COAD, COADREAD, KIRP, LIHC, LUADLUSC, and THCA from TCGA datasets. (**B**) The correlation between KIF3C expression and clinical characteristics in STAD, including the TNM-T stage, TNM-N stage, TNM-M stage, histologic stage, gender, age, H pylori infection and Barretts esophagus.

Due to the limited understanding of the clinical significance of KIF3C expression in pan-cancer prognosis, we conducted a comprehensive analysis to explore the relationship between KIF3C expression and clinicopathological characteristics in nine distinct cancer types, including BLCA, STAD, COAD, COADREAD, KIRP, LIHC, LUADLUSC, and THCA. As depicted in (Figure 3A), a statistically significant positive relationship was identified between KIF3C expression levels and tumor stage (P<0.05). These findings imply a plausible association between the expression of KIF3C and patient prognosis.

Furthermore, we extended our evaluation to investigate the relationship between KIF3C expression and clinical features in STAD. These analyses indicated a strong correlation between elevated KIF3C levels and pathologic T stage and patient age (Figure 3B), confirming the positive association between KIF3C expression and STAD progression. The summary of the correlation between KIF3C and clinical features in GC patients is presented in (Table 1), based on the TCGA-STAD cohort analysis.

**Table 1:** Features of the TCGA-STAD patients based on the KIF3C expression.

**Characteristic**	**Low expression of KIF3C**	**High expression of KIF3C**	**P-value**
n	187	188	
Pathologic T stage, n (%)			0.033
T1	14 (73.7%)	5 (26.3%)	
T2&T3&T4	169 (48.6%)	179 (51.4%)	
Pathologic N stage, n (%)			0.869
N0	57 (51.4%)	54 (48.6%)	
N1&N2&N3	124 (50.4%)	122 (49.6%)	
Pathologic M stage, n (%)			0.524
M0	167 (50.6%)	163 (49.4%)	
M1	11 (44%)	14 (56%)	
Pathologic stage, n (%)			0.318
Stage I	30 (56.6%)	23 (43.4%)	
Stage II&Stage III&Stage IV	147 (49.2%)	152 (50.8%)	
Histologic grade, n (%)			0.722
G1	4 (40%)	6 (60%)	
G2&G3	181 (50.8%)	175 (49.2%)	
Histological type, n (%)			0.453
Diffuse Type	32 (50.8%)	31 (49.2%)	
Mucinous Type	7 (36.8%)	12 (63.2%)	
Not Otherwise Specified	105 (50.7%)	102 (49.3%)	
Papillary Type	1 (20%)	4 (80%)	
Signet Ring Type	4 (36.4%)	7 (63.6%)	
Tubular Type	38 (55.1%)	31 (44.9%)	
Primary therapy outcome, n (%)			0.852
PD	29 (44.6%)	36 (55.4%)	
SD	7 (41.2%)	10 (58.8%)	
PR	2 (50%)	2 (50%)	
CR	114 (49.4%)	117 (50.6%)	
Residual tumor, n (%)			0.865
R0	149 (50%)	149 (50%)	
R1	8 (53.3%)	7 (46.7%)	
R2	9 (56.2%)	7 (43.8%)	
Anatomic neoplasm subdivision, n (%)			0.829
Fundus/Body	65 (50%)	65 (50%)	
Gastroesophageal Junction	22 (53.7%)	19 (46.3%)	
Other	3 (75%)	1 (25%)	
Stomach (NOS)	4 (66.7%)	2 (33.3%)	
Antrum/Distal	66 (47.8%)	72 (52.2%)	
Cardia/Proximal	24 (50%)	24 (50%)	
Reflux history, n (%)			0.143
No	85 (48.6%)	90 (51.4%)	
Yes	24 (61.5%)	15 (38.5%)	
Barretts esophagus, n (%)			0.319
No	103 (53.4%)	90 (46.6%)	
Yes	10 (66.7%)	5 (33.3%)	
H pylori infection, n (%)			0.712
No	82 (56.6%)	63 (43.4%)	
Yes	11 (61.1%)	7 (38.9%)	
Gender, n (%)			0.639
Female	69 (51.5%)	65 (48.5%)	
Male	118 (49%)	123 (51%)	
Race, n (%)			0.433
Asian	39 (52.7%)	35 (47.3%)	
Black or African American	5 (45.5%)	6 (54.5%)	
White	105 (44.1%)	133 (55.9%)	
Age, n (%)			0.011
<= 65	70 (42.7%)	94 (57.3%)	
> 65	116 (56%)	91 (44%)	

### The diagnostic value of KIF3C

**Figure 4:**
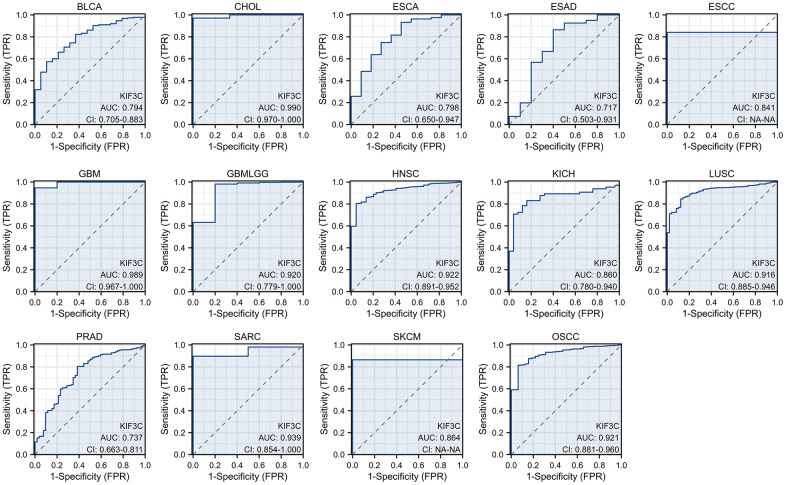
The area under the curve of ROC curves verified the diagnostic ability of KIF3C in BLCA, CHOL, ESCA, ESAD, ESCC, GBM, GBMLGG, HNSC, KICH, LUSC, PRAD, SARC, SKCM and OSCC.

The diagnostic accuracy of KIF3C was assessed using Receiver Operating Characteristic (ROC) curves. The AUC values from the ROC analysis indicate high diagnostic accuracy (AUC ≥0.9) in 7 cancer types, including CHOL (AUC=0.990), GBM (AUC=0.989), GBMLGG (AUC=0.920), HNSC (AUC=0.922), LUSC (AUC=0.916), SARC (AUC=0.939), and OSCC (AUC=0.921). In 7 cancer types, it exhibited a relative diagnostic accuracy ranging between 0.7 and 0.9, including BLCA (AUC=0.794), ESCA (AUC=0.798), ESAD (AUC=0.717), ESCC (AUC=0.841), KICH (AUC=0.860), PRAD (AUC=0.737) and SKCM (AUC=0.864) (Figure 4). An AUC greater than 0.9 indicates excellent discrimination. These findings suggest that KIF3C may serve as a promising diagnostic biomarker for various cancers.

### Functional enrichment analysis

**Figure 5:**
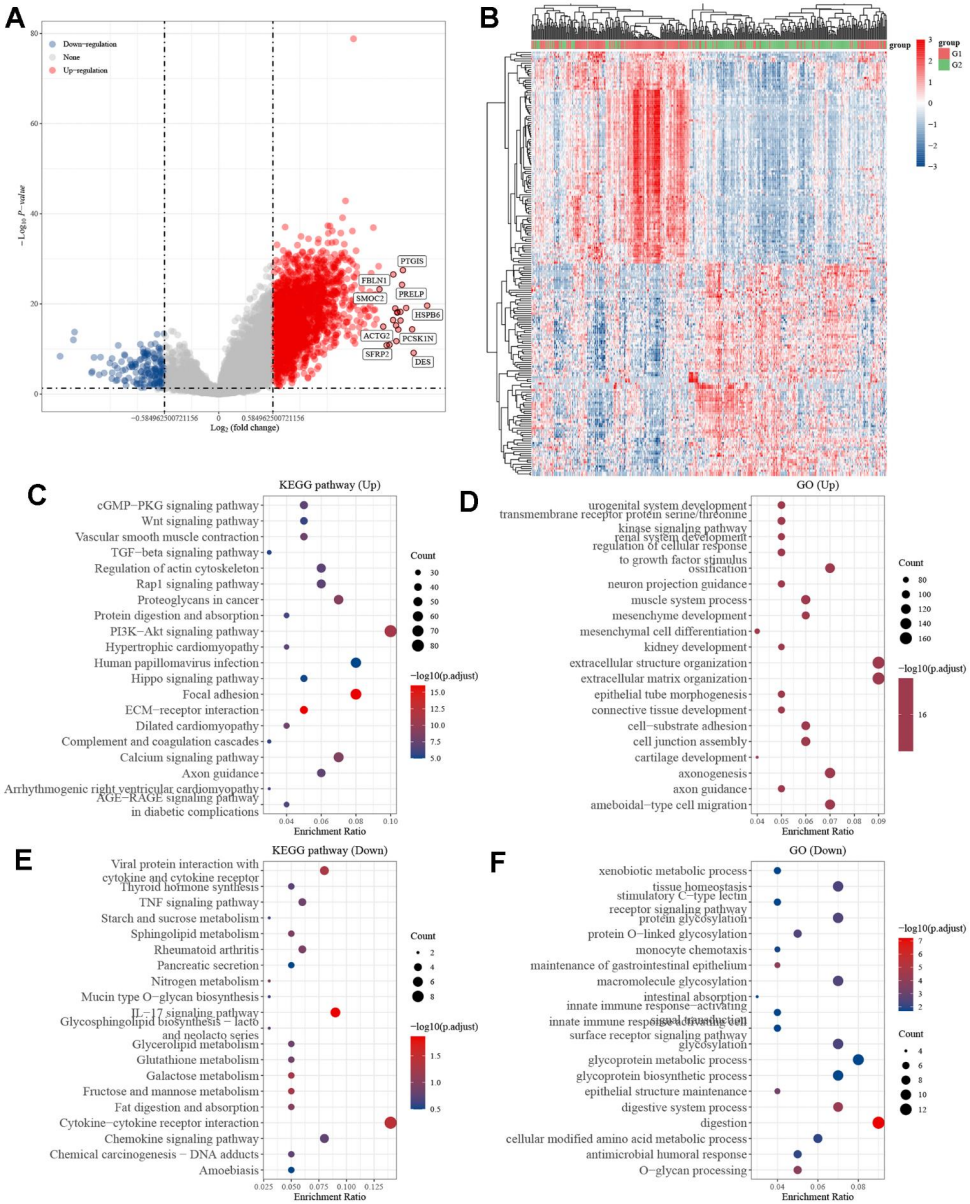
**Differentially expressed genes in STAD tissues with high and low KIF3C expression.** (**A**) Volcano map of differentially expressed genes. (**B**) Heatmap of differentially expressed genes. (**C**) KEGG and (**D**) GO enrichment analysis of differentially up-regulated genes. (**E**) KEGG and (**F**) GO enrichment analysis of differentially down-regulated genes.

Following a comprehensive examination of the gene expression profiles in the gastric cancer cohort from the TCGA database, we found a total of 2087 genes that exhibited differential expression between the high and low KIF3C expression groups. Of these genes, 1934 were found to be upregulated, while 153 were downregulated. To provide a visual representation of these differences, we created volcano plots and heatmaps (Figure 5A, 5B). Subsequently, we conducted KEGG pathway analysis focusing on the upregulated genes and GO enrichment analysis for the downregulated genes. The KEGG pathway analysis unveiled that KIF3C expression was linked to the PI3K-AKT signaling pathway (Figure 5C–5E). On the other hand, the GO function analysis demonstrated that KIF3C expression was associated with processes related to extracellular structure organization, extracellular matrix organization, and digestion (Figure 5D–5F). These findings shed light on potential pathways and biological processes influenced by KIF3C expression in gastric cancer.

### Analyses of immune infiltration, immune checkpoints, and immunotherapy response correlation between KIF3C and immune-related biomarker

**Figure 6:**
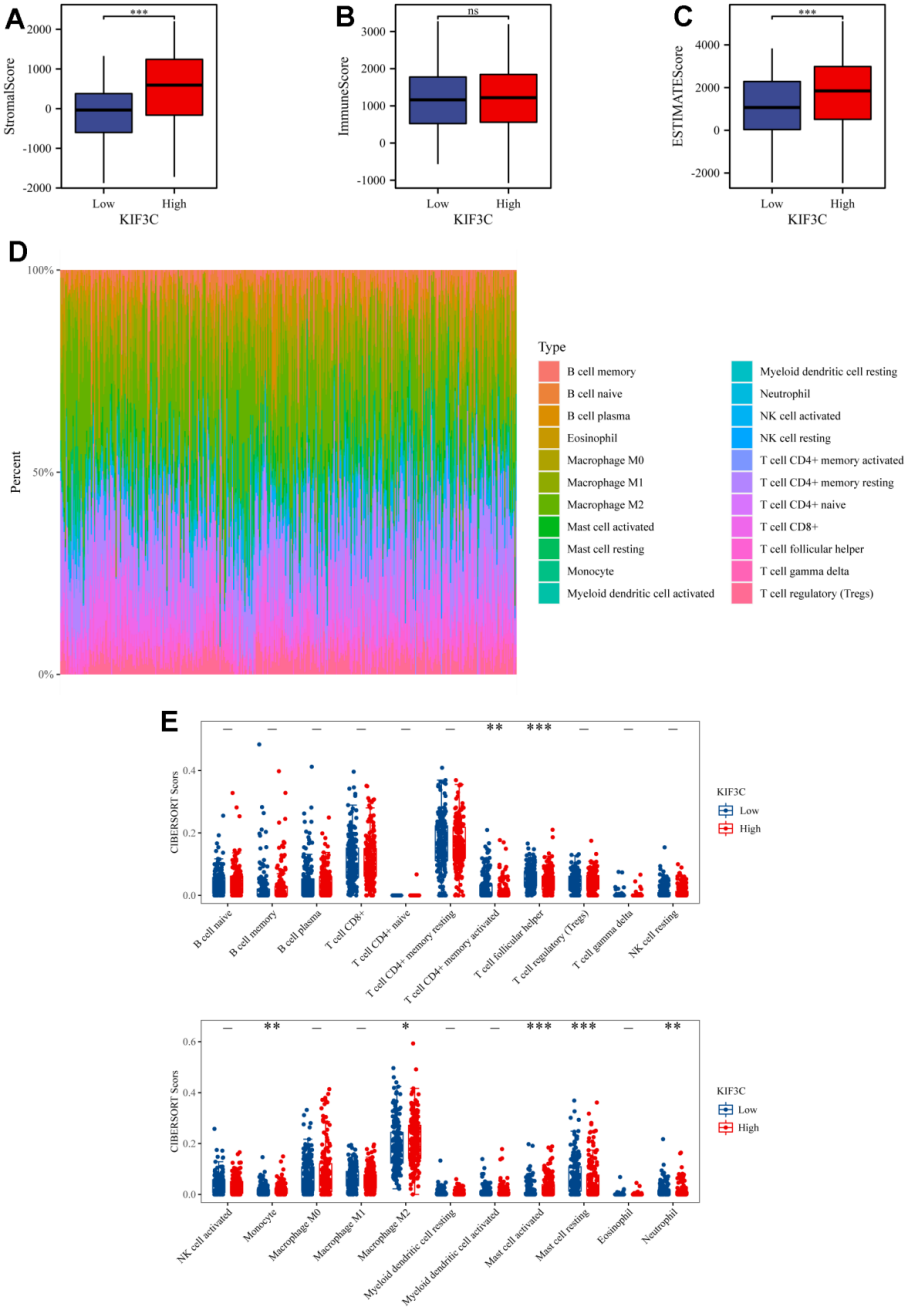
**ESTIMATE scores distribution and TIICs analysis by CIBERSORT.** (**A–C**) Distribution of ISs, SSs, and ESs between KIF3C high- and low-expression groups. (**D**) A bar chart showing the difference in the proportion of 22 TIICs in the TME of STAD. (**E**) A boxplot comparing the proportion of the 22 TIICs in the TME of STAD between the high- and low-expression groups (* p < 0.05, ** p < 0.01, *** p < 0.001; ns, not significant).

Graphs generated by the ESTIMATE algorithm unveil the relationship between KIF3C and immune infiltration scores. As depicted in (Figure 6A, 6C), the high-expression group displays a notably higher presence of SSs and ESs in contrast to the low-expression group. On the other hand, there is no notable difference in ISs between the high-expression and low-expression groups, as indicated in (Figure 6B). The distribution of 22 types of immune lymphocytes in the Tumor Microenvironment (TME) of STAD was assessed using the CIBERSORT algorithm (Figure 6D). Results of the immune landscape analysis between the high- and low-expression groups revealed that the proportions of Monocyte, Macrophage M2, and activated Mast cells were relatively higher in the KIF3C high-expression group compared to the KIF3C low-expression group. Conversely, infiltration levels of CD4+ activated memory T cells, T cell follicular helper, resting Mast cells, and Eosinophils were lower in the KIF3C high-expression group (Figure 6E).

**Figure 7:**
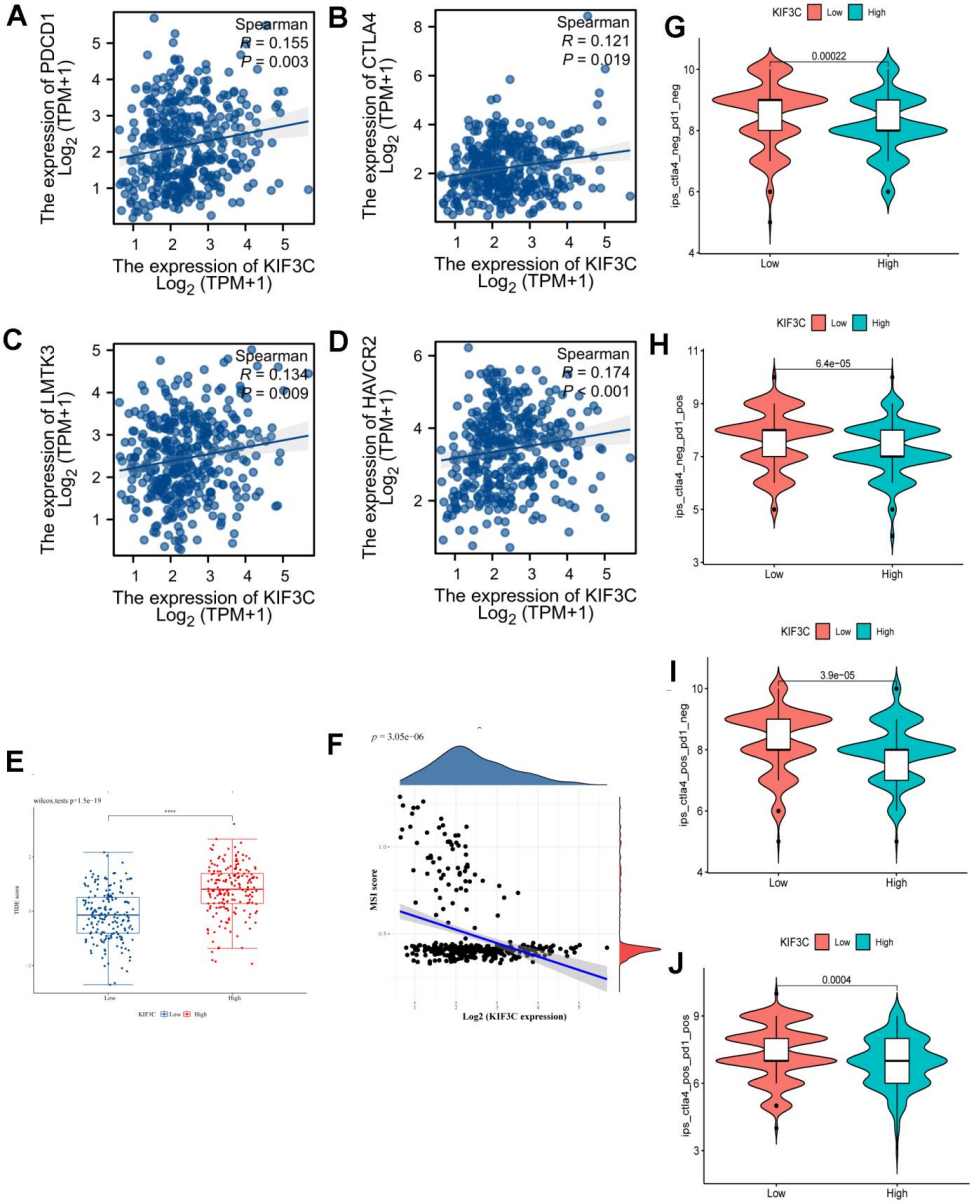
**Correlation analysis of KIF3C to the immune checkpoint, TIDE score, MSI, and IPS.** (**A**) PD-1, (**B**) CTLA4, (**C**) LMTK3, (**D**) HAVCR2. (**E**) TIDE score between KIF3C high- and low-expression expression groups. (**F**) MSI Score. (**G**) The IPS, (**H**) IPS-PD-1/PD-L1/PD-L2, (**I**) IPS-CTLA4, and (**J**) IPS-PD-1/PD-L1/PD-L2 + CTLA4.

We explored the association between immune checkpoints and KIF3C. The results indicate that KIF3C is positively associated with PD-1(PDCD1), CTLA4, LMTK3 and HAVCR2 (Figure 7A–7D). We examined the relationship between KIF3C expression and MSI, which hold relevance for immunotherapy response, across all tumors included in the TCGA dataset. KIF3C was negatively correlated with MSI in STAD (Figure 7F). Additionally, there was a significant correlation between TIDE score and high KIF3C expression level (Figure 7E). Considering the positive correlation of KIF3C with immune checkpoints, we subsequently explored the relationship between KIF3C and immune checkpoint inhibitors (ICIs). The Immune Profiling Score (IPS) comparison between high- and low-expression groups of KIF3C revealed that patients with high KIF3C expression exhibited a higher IPS for anti-PD-1 and anti-CTLA4 immunotherapy, implying a more favorable response to immunotherapy (Figure 7G–7J).

### Association analysis of KIF3C expression with drug sensitivity in STAD

**Figure 8:**
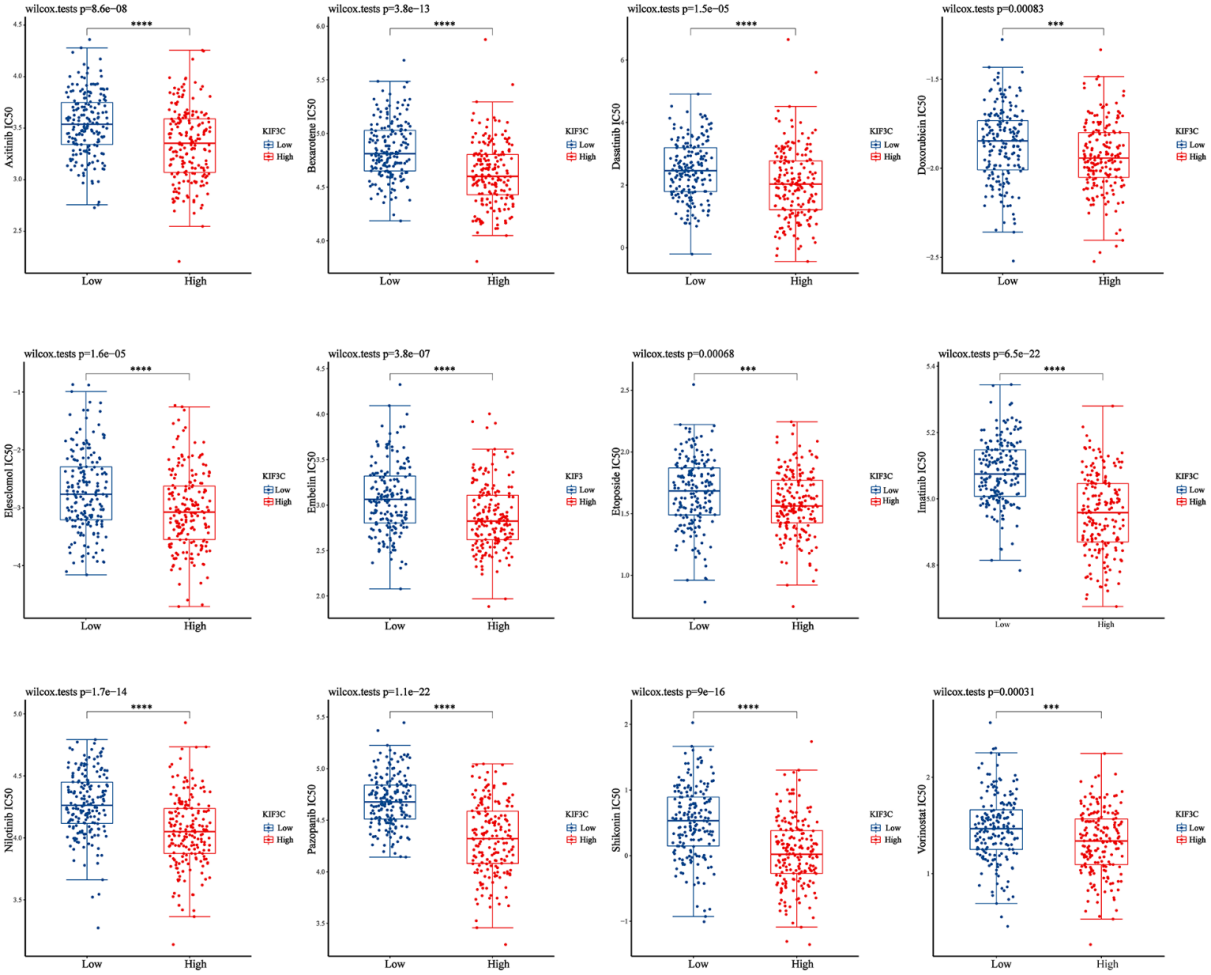
**Correction between KIF3C expression and drug sensitivity in gastric cancer.** *p < 0.05; **p < 0.01; ***p < 0.001.

We conducted a drug sensitivity analysis using the GDSC gene expression and drug sensitivity data as training data, along with the STAD-TCGA expression dataset as the test data. The KIF3C high-expression group and the KIF3C low-expression group were defined using the median KIF3C expression value as the cutoff. Subsequently, we compared the differences in drug sensitivity between these two groups. The results of this analysis were illustrated in (Figure 8), which indicate a significant disparity in drug sensitivity. Notably, in patients with stomach adenocarcinoma (STAD) who had higher KIF3C expression levels, there was increased sensitivity to specific drugs, including Axitinib, Bexarotene, Dasatinib, Doxorubicin, Elesclomol, Embelin, Etoposide, Imatinib, Nilotinib, Pazopanib, Shikonin, and Vorinostat. Our results suggest that KIF3C expression may influence the responsiveness of STAD patients to these drugs.

### Validation of KIF3C expression

**Figure 9:**
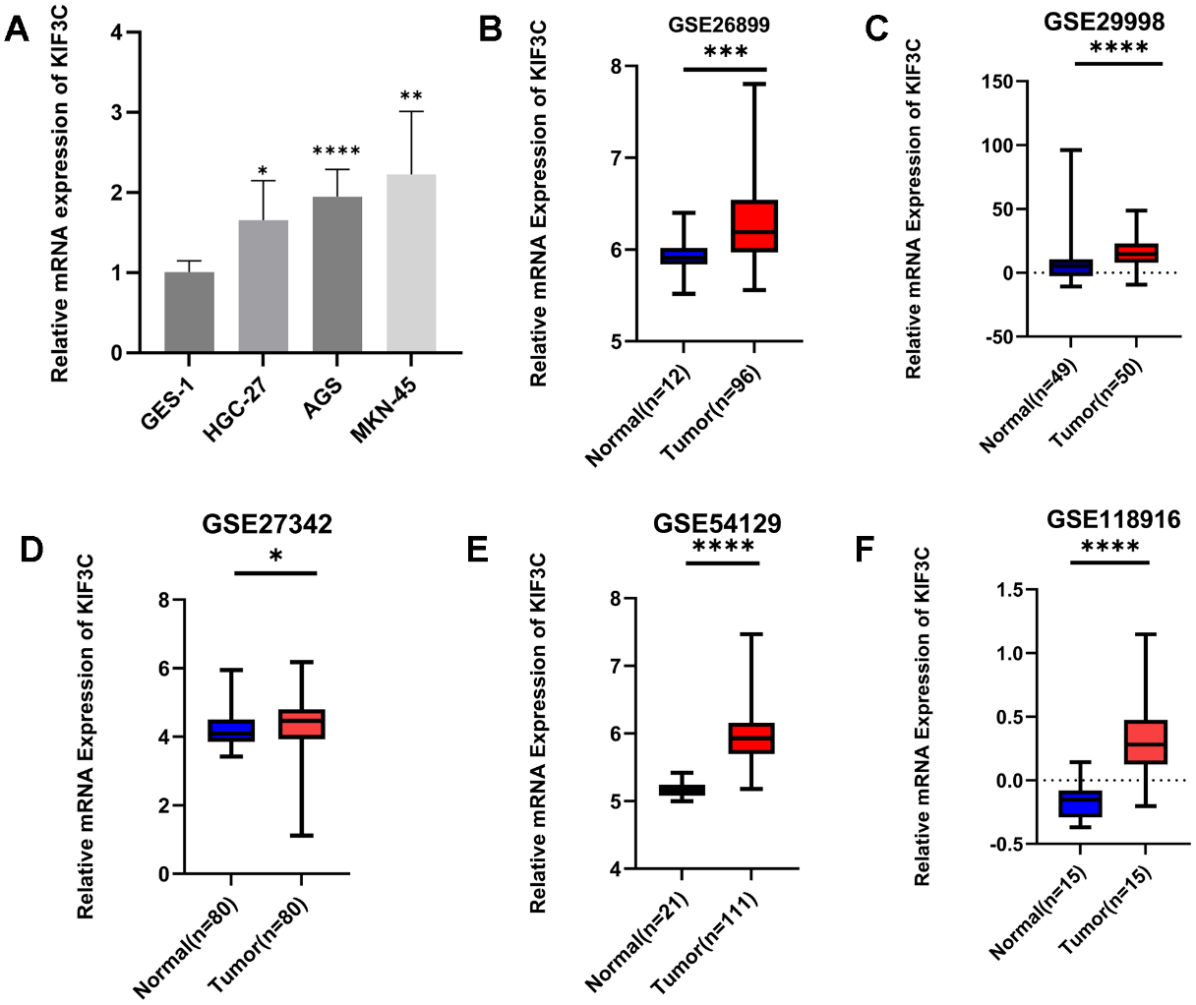
**Validation of KIF3C expression.** (**A**) qRT-PCR analysis of KIF3C mRNA in the GC cell lines and normal gastric cell line. KIF3C mRNA levels in GC tissues and normal tissues in (**B**) GSE26899, (**C**) GSE29998, (**D**) GSE27342, (**E**) GSE54129, (**F**) GSE118916. *p < 0.05, **p < 0.01, ***p < 0.001.

Our analysis revealed that KIF3C exhibited upregulation in gastric cancer (GC) samples when compared to normal gastric cell lines, as demonstrated by qPCR analysis (Figure 9A). Moreover, data from the GEO database supported these findings, demonstrating significantly elevated KIF3C expression in GC compared to normal samples (Figure 9B–9F). These cumulative findings offer robust evidence that KIF3C is indeed overexpressed in GC in contrast to normal samples.

### Knockdown of KIF3C suppresses the malignant phenotype of gastric cancer *in vitro*

**Figure 10:**
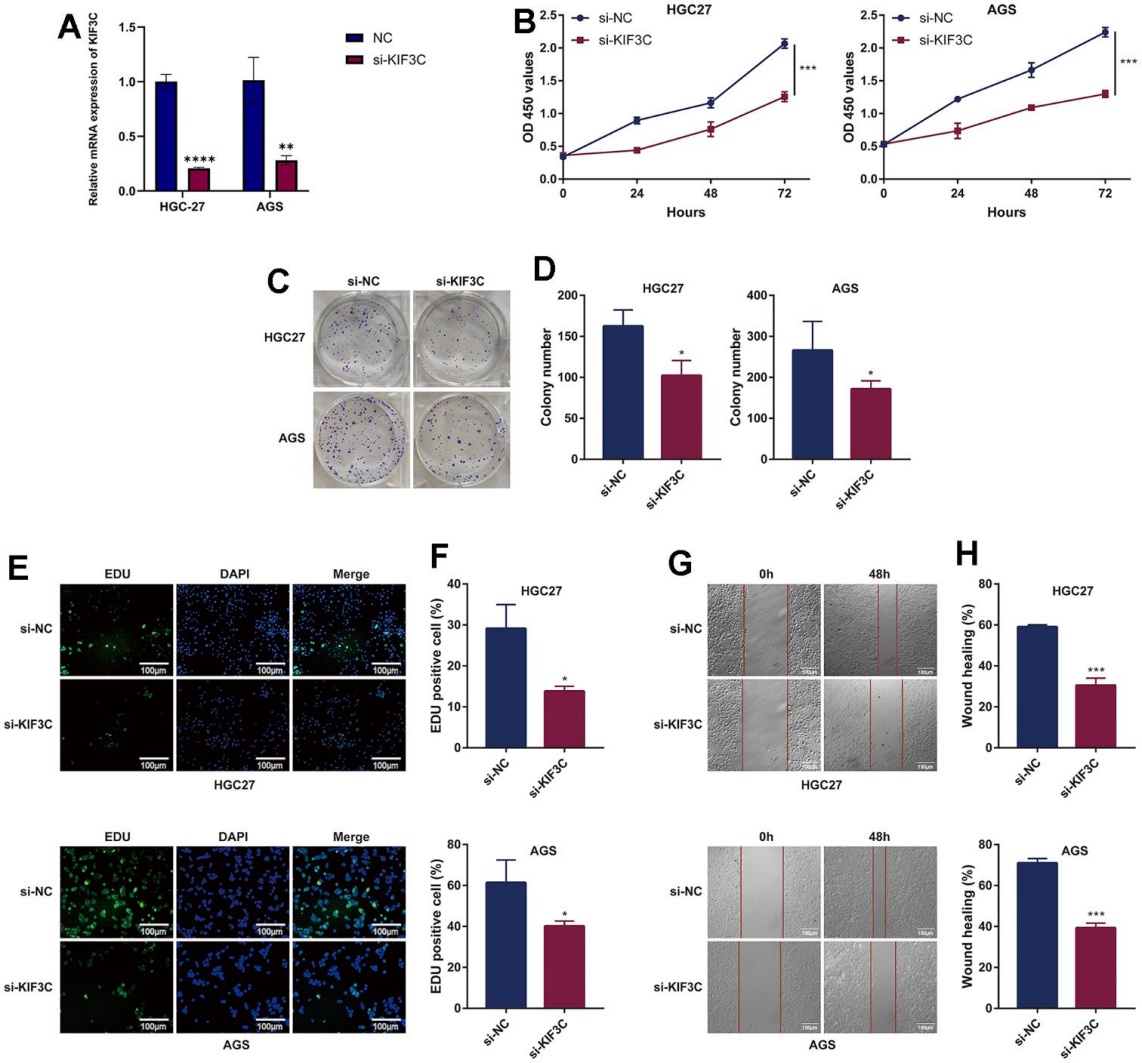
**KIF3C knockdown suppresses the malignant phenotype of GC cells.** (**A**) Knockdown efficiency of KIF3C in two Gastric cancer cell lines HGC-27 and AGS, including the si-NC and si-KIF3C groups. (**B**) CCK-8 assays were applied to detect the effect of KIF3C knockdown on the proliferation of HGC-27 and AGS cell lines. (**C**) Images of colony formation assay results after KIF3C knockdown in the HGC-27 and AGS cell lines. (**D**) Representative statistical analysis of the colony formation assay results. (**E**) DNA replication activity was assessed by an EdU-staining assay (green indicates the EdU-incorporated cells, blue indicates nuclei). GAPDH was used as an internal control. (**F**) Representative statistical analysis of the EdU assays results. (**G**) Wound-healing assays employed to detect the migration ability of KIF3C knockdown cells, including HGC-27 and AGS cells. (**H**) Representative statistical analysis of the wound-healing assays results. *p < 0.05; **p < 0.01; ***p < 0.001.

To acquire insights into the influence of KIF3C on the advancement of gastric cancer, we employed a method involving the suppression of KIF3C expression in gastric cancer cells by transfecting them with si-KIF3C. The downregulation of KIF3C in HGC-27 and AGS cells was confirmed following transfection with si-KIF3C, as compared to cells transfected with a control group (si-NC) (Figure 10A). Subsequently, we conducted CCK-8 assays to assess the influence of KIF3C on cell proliferation. The optical density (OD) values for HGC-27 and AGS cells, following transfection with si-KIF3C, exhibited a significant reduction compared to those cells transfected with si-NC, indicating a significant difference, as visually represented in (Figure 10B). To further validate these findings, we performed a colony development investigation. The colonies formed by HGC-27 and AGS cells transfected with si-KIF3C were considerably smaller in size compared to those formed by cells transfected with si-NC (Figure 10C, 10D). Moreover, we employed the EdU-staining assay to evaluate DNA replication activity, and the results showed a substantial reduction in EdU staining in HGC-27 and AGS cells following KIF3C suppression (Figure 10E, 10F). These findings collectively signify a substantial decrease in the growth of gastric cancer cells as a result of KIF3C knockdown.

**Figure 11:**
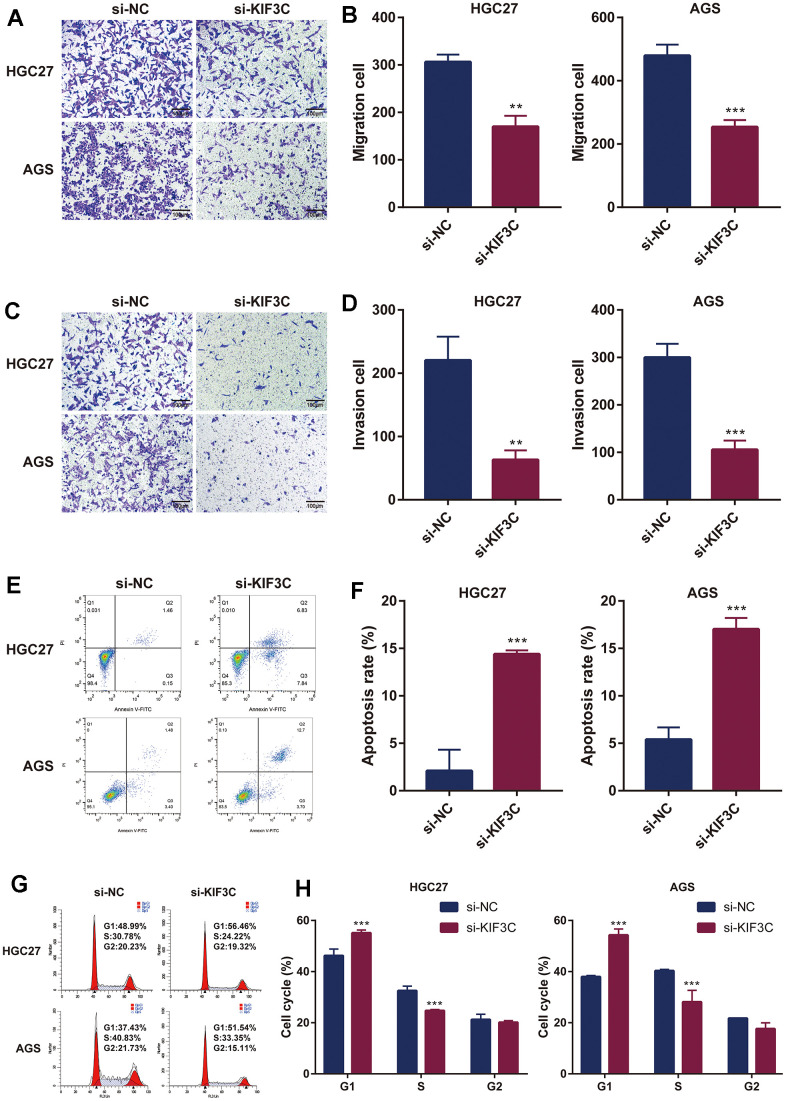
**KIF3C knockdown influences the biological functions in GC.** (**A**) Transwell migration assay of HGC-27 and AGS cells. (**B**) Representative statistical analysis of the transwell migration assays results. (**C**) Transwell invasion assay of HGC-27 and AGS cells. (**D**) Representative statistical analysis of the transwell invasion assays results. (**E**) Flow cytometry was used to detect apoptosis changes in HGC-27 and AGS. (**F**) Representative statistical analysis of the cell apoptosis assays results. (**G**) Flow cytometry was used to detect the cell cycle changes in HGC-27 and AGS. (**H**) Representative statistical analysis of the cell cycle assays results. *p < 0.05; **p < 0.01; ***p < 0.001.

Subsequently, we investigated the influence of KIF3C knockout on tumor cell migration through wound healing and transwell migration assays. The inhibition of wound healing was notably observed upon KIF3C silencing in HGC-27 and AGS cell lines (Figure 10G, 10H). Furthermore, the transwell assay distinctly demonstrated a significant suppression in the migratory capacity of both HGC-27 and AGS cells following KIF3C silencing (Figure 11A, 11B). These experiments revealed a considerably lower number of migratory cells in the si-KIF3C group as compared to HGC-27 and AGS cells transfected with si-NC, clearly indicating the role of KIF3C in promoting the migration capacity of GC cells. In the transwell invasion assay, the si-KIF3C group exhibited a significant decrease in invasion ability compared to si-NC, further confirming reduced cell invasion (Figure 11C, 11D). These results collectively suggest a significant enhancement of the invasion capacity of gastric cancer cells by KIF3C.

Subsequent flow cytometry analysis results illustrated that in HGC-27, the proportion of cells in the FITC-positive region in the KIF3C knockout group was 14.67%, significantly higher than the control group’s 1.61%. Similarly, in AGS cells, the proportion of cells in the FITC-positive region in the KIF3C knockout group was 16.40%, markedly higher than the 4.88% observed in the control group. These results indicated a greater incidence of apoptosis in KIF3C-siRNA-treated GC cells compared to those treated with scrambled siRNAs (Figure 11E, 11F). Given the observed effects of KIF3C on GC cell growth, to delve deeper into the cell cycle dynamics, we conducted a cell cycle assay, which elucidated an augmentation in the population of cells in the G1 phase and a concurrent reduction in the number of cells in the S phase subsequent to KIF3C knockdown in HGC-27 and AGS cell lines (Figure 11G, 11H).

## DISCUSSION

Gastric cancer (GC) is characterized by a high degree of heterogeneity and significantly impacts a large population annually. As diagnoses predominantly occur at advanced stages, coupled with elevated rates of chemotherapeutic resistance, there is a pressing need for expanded treatment options, particularly for patients with metastatic and unresectable GC. Despite the introduction of various targeted agents in GC, including Trastuzumab, Ramucirumab, and Pembrolizumab, few have demonstrated substantial benefits in terms of survival. This underscores the ongoing challenge of identifying newer and more effective therapeutic targets. In our investigation, we identified a notable upregulation of KIF3C in GC tissues, and this heightened expression correlated with advanced pathological stages and poorer survival outcomes in GC patients. These findings suggest a potential pivotal role for KIF3C in both the diagnosis and treatment of gastric cancer.

Our study initially procured pan-cancer data via the UCSC Xena platform to investigate the expression levels and prognostic significance of KIF3C. In comparison to normal tissues, KIF3C exhibited elevated expression across the majority of malignant tumors. Moreover, this heightened KIF3C expression was corroborated in gastric cancer cell lines and datasets. The variations in KIF3C expression among distinct tumor types may underscore diverse underlying functions and mechanisms. Subsequent survival analysis revealed that an elevated KIF3C is related to an unfavorable prognosis in gastric cancer. This suggests that, in contrast to other tumor types, KIF3C may exert a more substantial influence on the initiation and development of gastric cancer.

In our investigation, we found that the role of KIF3C in prostate cancer, non-small cell lung cancer (NSCLC), and breast cancer aligns with its function in gastric cancer. Surprisingly, in glioma, we observed an opposing effect of KIF3C compared to its role in gastric cancer. As for thyroid cancer, there haven’t been reports on the impact of KIF3C that we’ve come across. These diverse observations highlight the potential variability and complexity of KIF3C across different cancer types. We are committed to further exploring the specific mechanisms underlying KIF3C’s role in tumor development. We aim to comprehensively elucidate its differential effects across various cancer types in our future studies.

The kinesin superfamily proteins (KIFs) constitute a group of microtubule and ATP-dependent molecular motors, with the human genome containing 45 members categorized into 14 classes [13]. These proteins have been identified as playing pivotal roles in a variety of cellular processes, encompassing intracellular trafficking [14], mitosis, and cytokinesis [15], as well as in the context of birth defects [16]. Recently, there has been a burgeoning interest in investigating their functions in the realm of cancer progression [17]. Specifically regarding gastric cancer (GC), an array of KIF members, such as KIF5B [18], KIF1B [19], KIF14 [20], KIF11 [21], KIF15 [21], KIF18A [22], KIF20A [23], KIF21B [24], KIF22 [25], KIF23 [26], KIF26A [27], KIF26B [28], and more, have been recognized for their involvement in the progression of gastric cancer via various mechanisms. Moreover, agents aimed at KIFs have demonstrated substantial potential in the realm of cancer therapy. Among the Kinesin superfamily, the Kinesin-2 subfamily is of notable interest, comprising three members: KIF3A, KIF3B, KIF3C, and KIF17, all of which have been discovered in mice [29, 30]. KIF3A functions as a microtubule-directed motor subunit engaged in the subcellular transportation of the β-catenin-cadherin complex [31]. KIF3B, as a member of the Kinesin-2 subfamily, fulfills a pivotal function in the transportation of vesicles and membrane expansion throughout the process of cell mitosis [32]. KIF3C serves as a motor protein, actively engaging in axonal transport within neuronal cells [33], and it is prominently expressed within the central nervous system (CNS), where it significantly contributes to promoting axon growth and facilitating regeneration. This is achieved through its capacity to regulate and organize the microtubule cytoskeleton within the growth cone following injury [34]. On the contrary, KIF17 serves as a microtubule-based molecular motor, converting chemical energy through ATP hydrolysis into mechanical force, which is harnessed for cargo transport along microtubules [35]. The participation of the kinesin-2 subfamily in human tumors has been established, validating their influence on the malignant advancement of various tumor types, encompassing prostate cancer [36], liver cancer [37], and breast cancer [38]. In summary, the kinesin-2 subfamily, to varying degrees, is associated with the development of cancer. Although changes in the expression of driver proteins associated with cancer progression have been extensively investigated, the underlying mechanisms still require comprehensive elucidation. Previous evidence has revealed that members of the KIF family exert influence on the functionality of diverse human tumor cells via a range of signaling pathways. For example, KIF3A has been demonstrated to stimulate cell proliferation and invasiveness in advanced prostate cancer through its involvement in the Wnt signaling pathway [37].

Conversely, the suppression of KIF3C expression has been observed to hinder both tumor progression and metastasis in breast cancer, primarily through the inhibition of the TGF-β signaling pathway [39]. Moreover, it is firmly established that the PI3K/AKT pathway plays a central role in the regulation of tumor behavior across a spectrum of cancers, encompassing gastric cancer [40]. In this study, we conducted an analysis of enriched pathways, including the PI3K/AKT signaling pathway in gastric cancer. All these findings indicate the potential involvement of KIF3C in the PI3K/AKT pathway in the context of gastric cancer. However, it is essential to emphasize the need for additional research to validate this hypothesis.

In summary, our research findings reveal a significant relationship between KIF3C and the prognosis of gastric cancer patients, as well as the biology of cancer cells. Nevertheless, the precise role and associated mechanisms of KIF3C in the initiation and development of gastric cancer remain incompletely understood. Furthermore, a more comprehensive grasp of the regulatory mechanisms necessitates further investigation in animal models. We firmly believe that, with the refinement of related basic and clinical experiments, KIF3C holds the potential to emerge as a novel therapeutic target for gastric cancer, offering promise for improved outcomes among gastric cancer patients. Gastric cancer, characterized by a dismal prognosis among digestive tract tumors, underscores the critical importance of unraveling its underlying biological functions, as these factors significantly impact mortality rates. Targeted therapy has opened new avenues in the treatment of gastric cancer, with the quest for drugs targeting novel targets emerging as a recent research focal point. In light of this, our study has centered on exploring the influence of KIF3C on the biological functions of tumor cells and conducting related gene analyses. We envision that these findings will furnish invaluable insights and direction for the diagnosis and treatment of gastric cancer.

## CONCLUSIONS

Our research has demonstrated a notable connection between elevated KIF3C expression in gastric cancer (GC) and unfavorable clinicopathological features, as well as poorer patient survival. The multi-level evidence presented in our study underscores the significance of KIF3C in the development of GC and its potential as a biomarker for disease progression. We have elucidated the pivotal role of KIF3C as a regulator in cell proliferation, induction of apoptosis, and progression of the cell cycle. Furthermore, our findings indicate that interference with KIF3C can effectively impede GC growth by modulating cell cycle and apoptosis pathways. These findings posit KIF3C as a potentially auspicious target for the formulation of anti-tumor strategies within the domain of gastric cancer (GC).

## Supplementary Material

Supplementary Figure 1

## References

[1] Sung H, Ferlay J, Siegel RL, Laversanne M, Soerjomataram I, Jemal A, Bray F. Global Cancer Statistics 2020: GLOBOCAN Estimates of Incidence and Mortality Worldwide for 36 Cancers in 185 Countries. CA Cancer J Clin. 2021; 71:209–49. 10.3322/caac.21660 33538338

[2] GBD 2017 Stomach Cancer Collaborators. The global, regional, and national burden of stomach cancer in 195 countries, 1990-2017: a systematic analysis for the Global Burden of Disease study 2017. Lancet Gastroenterol Hepatol. 2020; 5:42–54. 10.1016/S2468-1253(19)30328-0 31648970 PMC7033564

[3] Chen Y, Jia K, Sun Y, Zhang C, Li Y, Zhang L, Chen Z, Zhang J, Hu Y, Yuan J, Zhao X, Li Y, Gong J, et al. Predicting response to immunotherapy in gastric cancer via multi-dimensional analyses of the tumour immune microenvironment. Nat Commun. 2022; 13:4851. 10.1038/s41467-022-32570-z 35982052 PMC9388563

[4] Vu HT, Zhang Z, Tehver R, Thirumalai D. Plus and minus ends of microtubules respond asymmetrically to kinesin binding by a long-range directionally driven allosteric mechanism. Sci Adv. 2022; 8:eabn0856. 10.1126/sciadv.abn0856 35417226 PMC9007332

[5] Hirokawa N, Tanaka Y. Kinesin superfamily proteins (KIFs): Various functions and their relevance for important phenomena in life and diseases. Exp Cell Res. 2015; 334:16–25. 10.1016/j.yexcr.2015.02.016 25724902

[6] Son HJ, Choi EJ, Yoo NJ, Lee SH. Somatic frameshift mutations of cancer-related genes KIF3C and BARD1 in colorectal cancers. Pathol Res Pract. 2019; 215:152579. 10.1016/j.prp.2019.152579 31400927

[7] Ma H, Zhang F, Zhong Q, Hou J. METTL3-mediated m6A modification of KIF3C-mRNA promotes prostate cancer progression and is negatively regulated by miR-320d. Aging (Albany NY). 2021; 13:22332–44. 10.18632/aging.203541 34537760 PMC8507285

[8] Liu H, Liu R, Hao M, Zhao X, Li C. Kinesin family member 3C (KIF3C) is a novel non-small cell lung cancer (NSCLC) oncogene whose expression is modulated by microRNA-150-5p (miR-150-5p) and microRNA-186-3p (miR-186-3p). Bioengineered. 2021; 12:3077–88. 10.1080/21655979.2021.1942768 34193018 PMC8806907

[9] Gao Y, Li L, Zheng H, Zhou C, Chen X, Hao B, Cao Y. KIF3C is associated with favorable prognosis in glioma patients and may be regulated by PI3K/AKT/mTOR pathway. J Neurooncol. 2020; 146:513–21. 10.1007/s11060-020-03399-7 32020481

[10] Gao Y, Zheng H, Li L, Zhou C, Chen X, Zhou X, Cao Y. KIF3C Promotes Proliferation, Migration, and Invasion of Glioma Cells by Activating the PI3K/AKT Pathway and Inducing EMT. Biomed Res Int. 2020; 2020:6349312. 10.1155/2020/6349312 33150178 PMC7603552

[11] Yao W, Jia X, Xu L, Li S, Wei L. MicroRNA-2053 involves in the progression of esophageal cancer by targeting KIF3C. Cell Cycle. 2021; 20:1163–72. 10.1080/15384101.2021.1929675 34057012 PMC8265789

[12] Yu G, Wang LG, Han Y, He QY. clusterProfiler: an R package for comparing biological themes among gene clusters. OMICS. 2012; 16:284–7. 10.1089/omi.2011.0118 22455463 PMC3339379

[13] Miki H, Setou M, Kaneshiro K, Hirokawa N. All kinesin superfamily protein, KIF, genes in mouse and human. Proc Natl Acad Sci USA. 2001; 98:7004–11. 10.1073/pnas.111145398 11416179 PMC34614

[14] Hirokawa N, Takemura R. Kinesin superfamily proteins and their various functions and dynamics. Exp Cell Res. 2004; 301:50–9. 10.1016/j.yexcr.2004.08.010 15501445

[15] Zhu C, Zhao J, Bibikova M, Leverson JD, Bossy-Wetzel E, Fan JB, Abraham RT, Jiang W. Functional analysis of human microtubule-based motor proteins, the kinesins and dyneins, in mitosis/cytokinesis using RNA interference. Mol Biol Cell. 2005; 16:3187–99. 10.1091/mbc.e05-02-0167 15843429 PMC1165403

[16] Kalantari S, Filges I. ‘Kinesinopathies’: emerging role of the kinesin family member genes in birth defects. J Med Genet. 2020; 57:797–807. 10.1136/jmedgenet-2019-106769 32430361 PMC7691813

[17] Chandrasekaran G, Tátrai P, Gergely F. Hitting the brakes: targeting microtubule motors in cancer. Br J Cancer. 2015; 113:693–8. 10.1038/bjc.2015.264 26180922 PMC4559828

[18] Wu ZW, Sha Y, Chen Q, Hou J, Sun Y, Lu WK, Chen J, Yu LJ. Novel intergenic KIF5B-MET fusion variant in a patient with gastric cancer: A case report. World J Clin Cases. 2021; 9:3350–5. 10.12998/wjcc.v9.i14.3350 34002144 PMC8107910

[19] Dong Z, Xu X, Du L, Yang Y, Cheng H, Zhang X, Li Z, Wang L, Li J, Liu H, Qu X, Wang C. Leptin-mediated regulation of MT1-MMP localization is KIF1B dependent and enhances gastric cancer cell invasion. Carcinogenesis. 2013; 34:974–83. 10.1093/carcin/bgt028 23354307

[20] Yang Z, Li C, Yan C, Li J, Yan M, Liu B, Zhu Z, Wu Y, Gu Q. KIF14 promotes tumor progression and metastasis and is an independent predictor of poor prognosis in human gastric cancer. Biochim Biophys Acta Mol Basis Dis. 2019; 1865:181–92. 10.1016/j.bbadis.2018.10.039 30404039

[21] Sun RF, He N, Zhang GY, Yu ZY, Li LS, Ma ZJ, Jiao ZY. Combined Inhibition of KIF11 and KIF15 as an Effective Therapeutic Strategy for Gastric Cancer. Curr Cancer Drug Targets. 2023; 23:293–306. 10.2174/1568009622666220616122846 35713129

[22] Wang L, Yang S, Sun R, Lu M, Wu Y, Li Y. [Expression of KIF18A in gastric cancer and its association with prognosis]. Zhonghua Wei Chang Wai Ke Za Zhi. 2016; 19:585–9. 27215532

[23] Sheng Y, Wang W, Hong B, Jiang X, Sun R, Yan Q, Zhang S, Lu M, Wang S, Zhang Z, Lin W, Li Y. Upregulation of KIF20A correlates with poor prognosis in gastric cancer. Cancer Manag Res. 2018; 10:6205–16. 10.2147/CMAR.S176147 30538567 PMC6260125

[24] Liu B, Qiang L, Guan B, Ji Z. Targeting kinesin family member 21B by miR-132-3p represses cell proliferation, migration and invasion in gastric cancer. Bioengineered. 2022; 13:9006–18. 10.1080/21655979.2022.2054755 35341446 PMC9161970

[25] Yu ZY, Jiang XY, Zhao RR, Qin JJ, Luo CJ, Ren YX, Ren W, Ma ZJ, Jiao ZY. Effect of KIF22 on promoting proliferation and migration of gastric cancer cells via MAPK-ERK pathways. Chin Med J (Engl). 2020; 133:919–28. 10.1097/CM9.0000000000000742 32187050 PMC7176455

[26] Liang WT, Liu XF, Huang HB, Gao ZM, Li K. Prognostic significance of KIF23 expression in gastric cancer. World J Gastrointest Oncol. 2020; 12:1104–18. 10.4251/wjgo.v12.i10.1104 33133380 PMC7579732

[27] Ma RR, Zhang H, Chen HF, Zhang GH, Tian YR, Gao P. MiR-19a/miR-96-mediated low expression of KIF26A suppresses metastasis by regulating FAK pathway in gastric cancer. Oncogene. 2021; 40:2524–38. 10.1038/s41388-020-01610-7 33674746

[28] Zhang H, Ma RR, Wang XJ, Su ZX, Chen X, Shi DB, Guo XY, Liu HT, Gao P. KIF26B, a novel oncogene, promotes proliferation and metastasis by activating the VEGF pathway in gastric cancer. Oncogene. 2017; 36:5609–19. 10.1038/onc.2017.163 28581513

[29] Malicki J, Besharse JC. Kinesin-2 family motors in the unusual photoreceptor cilium. Vision Res. 2012; 75:33–6. 10.1016/j.visres.2012.10.008 23123805 PMC3534980

[30] Yang Z, Roberts EA, Goldstein LS. Functional analysis of mouse kinesin motor Kif3C. Mol Cell Biol. 2001; 21:5306–11. 10.1128/MCB.21.16.5306-5311.2001 11463814 PMC87254

[31] Teng J, Rai T, Tanaka Y, Takei Y, Nakata T, Hirasawa M, Kulkarni AB, Hirokawa N. The KIF3 motor transports N-cadherin and organizes the developing neuroepithelium. Nat Cell Biol. 2005; 7:474–82. 10.1038/ncb1249 15834408

[32] Keil R, Kiessling C, Hatzfeld M. Targeting of p0071 to the midbody depends on KIF3. J Cell Sci. 2009; 122:1174–83. 10.1242/jcs.045377 19339549

[33] Sardella M, Navone F, Rocchi M, Rubartelli A, Viggiano L, Vignali G, Consalez GG, Sitia R, Cabibbo A. KIF3C, a novel member of the kinesin superfamily: sequence, expression, and mapping to human chromosome 2 at 2p23. Genomics. 1998; 47:405–8. 10.1006/geno.1997.5123 9480755

[34] Gumy LF, Chew DJ, Tortosa E, Katrukha EA, Kapitein LC, Tolkovsky AM, Hoogenraad CC, Fawcett JW. The kinesin-2 family member KIF3C regulates microtubule dynamics and is required for axon growth and regeneration. J Neurosci. 2013; 33:11329–45. 10.1523/JNEUROSCI.5221-12.2013 23843507 PMC6618697

[35] Wong-Riley MTT, Besharse JC. The kinesin superfamily protein KIF17: one protein with many functions. Biomol Concepts. 2012; 3:267–82. 10.1515/bmc-2011-0064 23762210 PMC3677786

[36] Liu Z, Rebowe RE, Wang Z, Li Y, Wang Z, DePaolo JS, Guo J, Qian C, Liu W. KIF3a promotes proliferation and invasion via Wnt signaling in advanced prostate cancer. Mol Cancer Res. 2014; 12:491–503. 10.1158/1541-7786.MCR-13-0418 24413182 PMC4001865

[37] Huang X, Liu F, Zhu C, Cai J, Wang H, Wang X, He S, Liu C, Yao L, Ding Z, Zhang Y, Zhang T. Suppression of KIF3B expression inhibits human hepatocellular carcinoma proliferation. Dig Dis Sci. 2014; 59:795–806. 10.1007/s10620-013-2969-2 24368420 PMC3958719

[38] Li T, Zhai D, Zhang M, Ye R, Kuang X, Shao N, Bi J, Lin Y. KIF17 maintains the epithelial phenotype of breast cancer cells and curbs tumour metastasis. Cancer Lett. 2022; 548:215904. 10.1016/j.canlet.2022.215904 36089118

[39] Wang C, Wang C, Wei Z, Li Y, Wang W, Li X, Zhao J, Zhou X, Qu X, Xiang F. Suppression of motor protein KIF3C expression inhibits tumor growth and metastasis in breast cancer by inhibiting TGF-β signaling. Cancer Lett. 2015; 368:105–14. 10.1016/j.canlet.2015.07.037 26272184

[40] Fattahi S, Amjadi-Moheb F, Tabaripour R, Ashrafi GH, Akhavan-Niaki H. PI3K/AKT/mTOR signaling in gastric cancer: Epigenetics and beyond. Life Sci. 2020; 262:118513. 10.1016/j.lfs.2020.118513 33011222

