# Supplementary figures and images for: KIF3C: an emerging biomarker with prognostic and immune implications across pan-cancer types and its experiment validation in gastric cancer
Source: Aging (Albany NY). 2024 Mar 28;16(7):6163–87. doi: 10.18632/aging.205694 (PMC11042961; doi:10.18632/aging.205694)

## SUPPLEMENTARY FIGURE

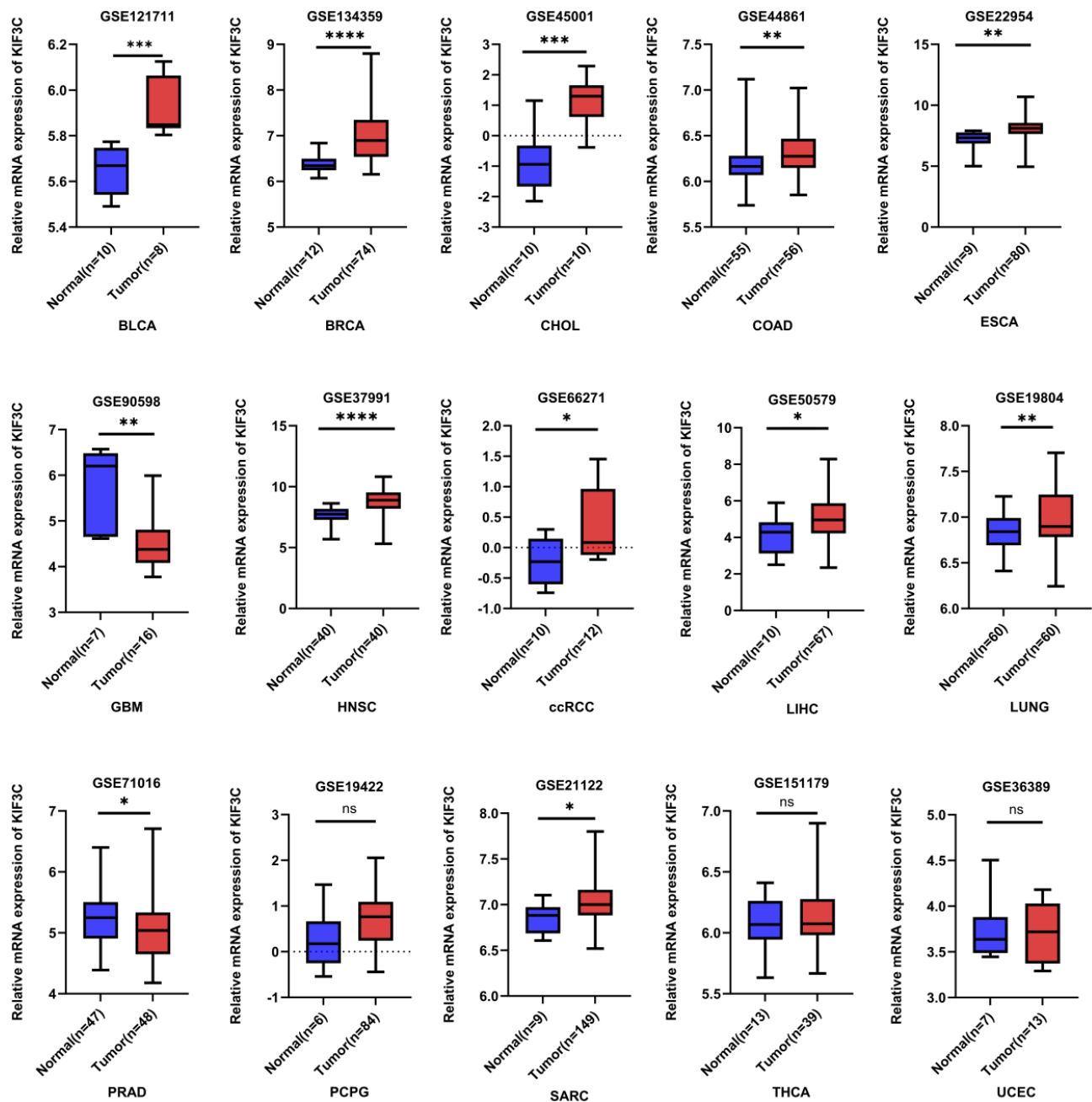

Supplementary Figure 1. The box plots of KIF3C expression levels in GEO.

Supplement: Supplementary Figure 1 [file aging-16-205694-s001.pdf]
